# Observation d'un cas index d'hypoparathyroïdie familiale autosomique dominante révélée par une hypocalcémie récidivante: à propos d'un cas

**DOI:** 10.11604/pamj.2026.53.40.50439

**Published:** 2026-01-28

**Authors:** Djikoldinguem Marschall Mouandilmadji, Mbaye Sene, Abdoulaye Dieme, Mouhamed Mbar Niang, Kawtar Jamil, Harine Abdel Aziz Garba, Ngone Diaba Diack, Nafy Ndiaye, Abdoulaye Leye

**Affiliations:** 1 Service d'Endocrinologie-Diabétologie, Centre Hospitalier Universitaire de Référence Nationale, N'Djaména, Tchad,; 2 Conseil National de Développement de la Nutrition, Dakar, Sénégal,; 3 Service d'Endocrinologie-Diabétologie-Nutrition, Centre Hospitalier National de Pikine, Dakar, Sénégal,; 4 Service de Médecine Interne, Hôpital de la Paix de Ziguinchor, Ziguinchor, Sénégal,; 5 Service de Médecine Interne, Hôpital Régional Amadou Tidiane Ba, Sédhiou, Sénégal,; 6 Service de Médecine Interne, Centre Hospitalier Universitaire de Référence Nationale, N'Djaména, Tchad

**Keywords:** Hypocalcémie, hypoparathyroïdie primaire, CaSR, transmission autosomique dominante, cas clinique, Hypocalcemia, primary hypoparathyroidism, CaSR, autosomal dominant inheritance

## Abstract

L'hypoparathyroïdie primaire est une affection rare responsable d'une hypocalcémie récurrente, dont l'identification peut conduire au dépistage familial. Elle peut se manifester par des symptômes cliniques sensitifs et neuromusculaires pouvant à l'extrême engager le pronostic vital, comme elle peut être asymptomatique de découverte fortuite. Ses étiologies sont multiples et la difficulté réside dans la recherche génétique. Le traitement doit être à la fois symptomatique et étiologique. Nous rapportons le cas d'une patiente de 42 ans présentant une hypocalcémie récidivante associée à une hypoparathyroïdie (PTH) basse, ce qui a permis de diagnostiquer une hypoparathyroïdie familiale chez deux de ses filles asymptomatiques. La transmission autosomique dominante est fortement suggérée. Ce cas illustre la nécessité du dépistage biologique des apparentés et l'importance croissante de la génétique dans l'étiologie des hypoparathyroïdies primaires.

## Abstract (en)

Primary hypoparathyroidism is a rare disorder responsible for recurrent hypocalcemia, the identification of which may lead to family screening. It can present with sensory and neuromuscular clinical manifestations that may, in severe cases, be life-threatening, or it may remain asymptomatic and be discovered incidentally. Its etiologies are diverse, and the main challenge lies in genetic investigation. Management must be both symptomatic and etiological. We here report the case of a 42-year-old woman presenting with recurrent hypocalcemia associated with low parathyroid hormone (PTH) levels, which led to the diagnosis of familial hypoparathyroidism in two of her asymptomatic daughters. Autosomal dominant inheritance is strongly suggested. This case highlights the need for biological screening of relatives and the growing importance of genetics in the etiology of primary hypoparathyroidism.

## Introduction

L'hypoparathyroïdie primaire est une affection endocrine rare, secondaire à une sécrétion insuffisante de PTH par les glandes parathyroïdiennes en l'absence de chirurgie cervicale [1]. Elle peut se révéler à tout âge, du nouveau-né à l'adulte, et se manifeste essentiellement par les symptômes d'une hypocalcémie. Les manifestations les plus fréquemment observées sont les manifestations neuromusculaires (paresthésies, crampes, tétanie) [2]. Le diagnostic est évoqué devant l'association biologique d'une hypocalcémie, d'une hyperphosphorémie et d'une concentration sanguine de PTH inappropriée nulle ou basse ou dans l'intervalle des valeurs normales, et ce, constaté au moins sur deux dosages à 15 jours d'intervalle. Devant une hypocalcémie récidivante et isolée, l'hypoparathyroïdie familiale doit être envisagée. Les mutations activatrices du récepteur sensible au calcium (CaSR) constituent une étiologie fréquente dans les formes autosomiques dominantes. L'avancée des tests génétiques a mis en évidence le rôle majeur des mutations du récepteur sensible au calcium (CaSR), notamment dans les formes autosomiques dominantes. Nous rapportons ici un cas d'hypocalcémie récidivante chez une adulte, ayant conduit au dépistage familial de premier degré et à la mise en évidence de plusieurs autres cas asymptomatiques. Cette observation illustre l'intérêt clinique du dépistage biologique des apparentés, particulièrement dans les contextes où l'accès aux tests génétiques demeure limité.

## Patient et observation

**Informations sur la patiente:** une patiente de 42 ans sans antécédents de chirurgie cervicale ni de radiothérapie cervicale consulte pour des paresthésies, crampes, fatigabilité à l'effort. Depuis 6 ans, elle présente des épisodes récidivants d'hypocalcémie ayant nécessité plusieurs évaluations dans différents centres de santé et des mises sous calcithérapie ponctuelles. Il n'existe pas d'antécédents familiaux connus de troubles phosphocalciques au moment de l'admission.

**Résultats cliniques:** à l'examen clinique, il n'existe aucune cicatrice cervicale. On note une sécheresse cutanée, une tétanie, un signe de Chvostek et de Trousseau positif, une absence de signes extrapyramidaux, une absence de crises comitiales et l’examen des autres appareils était sans anomalies.

**Chronologie de l'épisode en cours:** il y a 6 ans: début d'épisodes d'hypocalcémie récidivants, traités ponctuellement par calcium. Derniers mois, aggravation de la symptomatologie: paresthésies, crampes, fatigabilité. Jour de la consultation: découverte d'une hypocalcémie sévère et d'une PTH basse. Après le bilan: orientation vers une hypoparathyroïdie primaire d'allure génétique. Après enquête familiale: deux filles diagnostiquées asymptomatiques. À 3 mois de traitement: normalisation de la calcémie, disparition des symptômes.

**Évaluation diagnostique:** le bilan biologique initial retrouve une hypocalcémie à 51,9 mg/l, une hyperphosphatémie à 84 mEq/l et un effondrement du PTH intact à moins de 6 pg/ml. Le dosage sanguin de la vitamine D et du magnésium était normal avec des valeurs respectives de 42,1 µg/l et 19 mg/l. La calciurie était normale avec une phosphaturie de 24h basse à 0,27 g/24h. L’immunologie était négative (anticorps anti-nucléaires et auto-anticorps anti-CasR). La recherche d'insuffisance surrénalienne était négative et de candidose en faveur d'un APECED négatif. Chez deux filles (6 ans et 4 ans): l’hypocalcémie, l’hyperphosphatémie et la PTH étaient intactes et basses en faveur d'une hypoparathyroïdie primaire familiale (Tableau 1).

**Tableau 1:** données biologiques de la famille

Famille	Mère (cas index)	Mère (cas index)	Fille 1	Fille 2
Calcémie mg/L	58,3	51,9	64	59
Phosphorémie mEq/L	78,66	84	72	80
PTH intacte pg/mL	10,63	6	11,4	14,5
Vitamin D mg/L	40,03	42,1	38	40
Magnésémie mg/L	19	19	Non faite	Non faite
Calciurie des 24h en g/24h	74,4	74,4	Non faite	Non faite

Mère (cas index): premier bilan biologique puis contrôle dans un intervalle de 15 jours. Fille 1: sa fille ainée âgée de 6 ans. Fille 2: la cadette âgée de 4 ans

**Figure 1:**
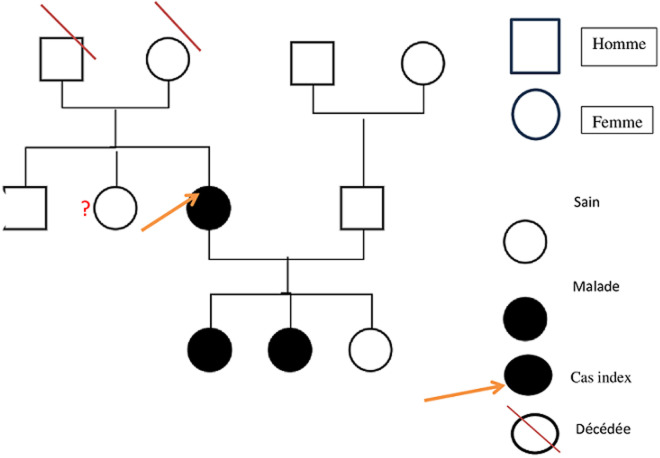
arbre généalogique

**Diagnostic:** hypoparathyroïdie primaire familiale, probablement autosomique dominante, évoquée devant: une hypocalcémie sévère persistante, une hyperphosphatémie, une PTH inappropriée basse, une calciurie normale, l'atteinte de deux enfants sur trois (Figure 1).

**Interventions thérapeutiques:** la patiente a été mise sous: supplémentation orale en calcium et vitamine D active selon le protocole de correction de l'hypoparathyroïdie.

**Suivi et résultats des interventions:** après 3 mois de traitement régulier, nous constatons la correction des signes cliniques et la normalisation de la calcémie à 74,9 mg/l mais la PTH intacte est toujours indétectable. Les deux filles ont été orientées pour une prise en charge spécialisée et une analyse génétique familiale est prévue mais non disponible encore dans notre région.

**Point de vue de la patiente:** la patiente rapporte un «mieux-être rapide», la disparition des crampes, et une amélioration notable de sa qualité de vie. Elle exprime un soulagement d'avoir enfin un diagnostic clair après plusieurs années d'errance médicale et se dit rassurée concernant la prise en charge de ses enfants.

**Consentement éclairé:** la patiente a donné son consentement éclairé pour la publication de ce cas clinique et l'utilisation des données familiales à des fins scientifiques, dans le respect de l'anonymat.

## Discussion

L'hypocalcémie est une anomalie métabolique fréquente dont la sévérité varie de formes asymptomatiques à des crises tétaniques mettant en jeu le pronostic vital [3]. Les manifestations les plus typiques incluent paresthésies, crampes, spasmes musculaires, tétanie, engourdissements péribuccaux et convulsions. Dans notre série, seule la patiente index présentait une symptomatologie neuromusculaire marquée, tandis que les deux autres cas ont été identifiés par dépistage familial [4]. Les formes familiales d'hypoparathyroïdie, souvent sous-diagnostiquées, pourraient être plus fréquentes qu'estimées, notamment dans les pays à ressources limitées. L'hypoparathyroïdie primaire liée à des mutations génétiques est beaucoup plus rare que les formes auto-immunes ou post-chirurgicales, elle représente environ 3 à 5% de l'ensemble des cas d'hypoparathyroïdie primaire [5]. L'hypoparathyroïdie génétique autosomique dominante liée à des mutations activatrices du CaSR représente une cause classique des hypocalcémies chroniques avec PTH basse et calciurie normale ou élevée.

Dans les hypocalcémies persistantes associées à une PTH basse, une hyperphosphorémie et une absence d'antécédent chirurgical cervical, l'hypoparathyroïdie primaire doit être fortement suspectée. Le diagnostic repose sur l'association d'une calcémie basse durable, d'une PTH inappropriée et d'une calciurie souvent normale, particulièrement suggestive d'une mutation activatrice du CaSR [6]. La présence de plusieurs cas au sein d'une même fratrie, avec transmission verticale, évoque un mode de transmission autosomique dominant. Dans les contextes où les tests génétiques ne sont pas disponibles, un dépistage biologique ciblé des apparentés de premier degré demeure indispensable. Malgré les progrès de la biologie moléculaire, un pourcentage notable d'hypoparathyroïdies demeure sans diagnostic étiologique clair [7]; les panels de gènes et le séquençage à haut débit devraient permettre d'améliorer cette identification [8]. Certaines complications, notamment oculaires comme la cataracte observée chez notre première patiente, ainsi que rénales ou neurologiques, doivent être activement recherchées.

Le traitement de l'hypoparathyroïdie repose principalement sur la calcithérapie orale (40 à 60 mg/kg/j de calcium élément en deux prises associées à une supplémentation en dérivés 1-alpha hydroxylés de la vitamine D). La posologie de la vitamine D est ensuite ajustée afin d'obtenir une calcémie > 2,2 mmol/L tout en maintenant une calciurie < 5 mg/kg/j [9], avec une réduction progressive des doses lorsque l'équilibre est atteint. Une alternative thérapeutique prometteuse est la PTH recombinante, administrée en injections fractionnées ou en perfusion continue, permettant une meilleure stabilité de la calcémie et un contrôle optimal de la calciurie, tout en réduisant le risque de lithiases et de néphrocalcinose [10]. Son utilisation reste limitée par son coût et sa disponibilité. Dans notre observation, seule la patiente index a reçu une vitamine D, tandis qu'une surveillance simple a suffi pour les deux autres patientes asymptomatiques.

Le pronostic des hypoparathyroïdies génétiques dépend largement de la précocité du diagnostic et de la qualité du suivi. Après un an d'évolution, aucune de nos patientes n'a présenté de complication rénale (Tableau 2), témoignant d'un bon contrôle métabolique. L'identification précoce des cas index, suivie d'un dépistage familial systématique, demeure essentielle pour prévenir les complications aiguës (tétanie, convulsions) et chroniques (calculs rénaux, cataracte, atteinte cérébrale). Notre observation souligne également les défis rencontrés dans les pays à ressources limitées, où l'accès restreint à la biologie moléculaire limite le diagnostic étiologique. L'intégration progressive des outils génétiques, notamment les panels de gènes et le séquençage NGS, constitue une étape clé pour améliorer la prise en charge et affiner le pronostic des patients présentant une hypoparathyroïdie primaire.

**Tableau 2:** données biologiques et évolutives des patientes

Famille	Calcium en mg/L	Phosphore en mEq/L	Suivi après 1 an	Calcium en mg/L	Phosphore en mEq/L
Mère (cas index	51,9	84		80	53
Fille 1	64	72		99	53
Fille 2	59	80		115	50

## Conclusion

Dans notre observation, l'hypoparathyroïdie primaire a pu être diagnostiquée aisément grâce à l'association d'une hypocalcémie persistante, d'une PTH inappropriée et d'une calciurie normale, mais l'identification de la cause exacte a été limitée par l'absence d'accès aux études génétiques, pourtant essentielles pour confirmer notamment une mutation activatrice du récepteur calcique, étiologie fortement suspectée ici du fait du caractère familial et de la transmission verticale. Le dépistage des apparentés de premier degré s'est révélé déterminant pour identifier deux cas supplémentaires asymptomatiques, soulignant l'importance d'un repérage précoce des cas index dans les contextes à ressources limitées. La prise en charge a reposé sur une supplémentation en calcium et en dérivé 1α-hydroxylé de la vitamine D chez la patiente symptomatique, avec adaptation des doses selon la calcémie et la calciurie, tandis que les autres patientes ont bénéficié d'une simple surveillance.
